# Magnitude and determinants of iron deficiency anemia among first antenatal care attending pregnant women at Addis Ababa, Ethiopia

**DOI:** 10.1371/journal.pone.0355295

**Published:** 2026-08-05

**Authors:** Kedir Dendir, Desalegn Fente, Zemenu Tamir, Moges Wordofa, Abush Getaneh, Tigist Getahun, Mehari Meles, Chala Besha, Wossene Habtu, Yoseph Tolcha

**Affiliations:** 1 St. Paul’s Hospital Millennium Medical College, Addis Ababa, Ethiopia; 2 Department of Medical Laboratory Sciences, College of Health Sciences, Addis Ababa University, Addis Ababa, Ethiopia; 3 Departments of Medical Laboratory Science, College of Health Science, Dilla University, Dilla, Ethiopia; 4 Department of Medical Laboratory Sciences, Ethiopian Public Health Institute, Addis Ababa, Ethiopia; Para Federal University, BRAZIL

## Abstract

Iron deficiency anemia during pregnancy is a major global health concern, responsible for approximately 50% of anemia cases worldwide. A prospective, institution-based, cross-sectional study was conducted from April to May 2024 among 170 first antenatal care attendees at Selam Health Centre in Addis Ababa, Ethiopia. Structured questionnaires were used to collect demographic and pregnancy-related data, and blood samples were taken to measure ferritin and hemoglobin levels using specific analyzers, following ethical consent. Data were analyzed using SPSS version 27, with factors associated with iron deficiency anemia assessed through multivariate logistic regression, considering a p-value below 0.05 as statistically significant. The assessment documented in this research determined that the prevalence of anemia was determined 9.4% (16/170) have a mean Hgb level <11g/dl of, with a specific focus on iron deficiency anemia, which affected 4.1%(7/170) of the participants which have a ferritin level <15µg/l. Iron-deficiency anemia was significantly associated with lower mid-upper arm circumference (AOR = 8.33, 95% CI; 1.23-56.33, P = 0.03), less meal frequency per day (AOR = 5.53, 95% CI; 1.83–36.72, P = 0.046), and dietary diversity score less than five (AOR=7.12, 95% CI; 1-50.71, P = 0.04). In conclusion, our investigation revealed a low prevalence of iron deficiency anemia. Despite this, factors such as low mid-upper arm circumference, poor dietary diversity, and reduced meal frequency were noted. We recommend educating pregnant mothers on nutrition, and conducting community-based research to further understand these factors.

## Introduction

Iron is essential for hemoglobin synthesis, enabling oxygen transport. It undergoes absorption, transport, utilization, and storage, with losses balanced by intestinal absorption. Most iron recycling occurs in the spleen, supporting red blood cell production [1,2].

Iron deficiency anemia (IDA) arises from poor intake, absorption, or blood loss, affecting maternal health, especially during pregnancy when iron needs increase. Hepcidin regulates iron metabolism, and imbalances can lead to anemia. IDA symptoms include fatigue, weakness, and cognitive issues, impacting maternal and fetal well-being [3,4].

Iron deficiency anemia (IDA) is a leading global health concern for women, affecting over two billion people, especially pregnant women. It accounts for nearly 50% of anemia cases worldwide. IDA prevalence varies, with higher rates in low-income countries compared to industrialized nations [5]. In Ethiopia, anemia affects 29% of pregnant women, with IDA contributing significantly [6]. Poor iron stores at conception and increased pregnancy demands exacerbate the issue. This study aims to address gaps in lack of a recent data, assess IDA prevalence, and identify contributing factors to improve maternal care and health policies. Furthermore, most previous studies have not adequately addressed the sociodemographic determinants of IDA in urban health centers such as Selam Health Center, Addis Ababa [7–9]. This study therefore aims to fill this gap by examining both the magnitude and determinants of IDA among first ANC attendees.

Most investigations have focused on general antenatal populations or trimester‑based groups, with limited attention to women at their first ANC visit who provide baseline data before supplementation or interventions [8,9]. Furthermore, many reports rely solely on hemoglobin levels, whereas our study incorporates multiple hematological and nutritional measures (Hb, Ferritin, CRP, MUAC, and DDS) to more accurately capture iron deficiency anemia rather than general anemia [7]. Finally, while national prevalence data exist, localized evidence from Addis Ababa remains scarce, despite urban centers having distinct dietary patterns, healthcare access, and socioeconomic conditions compared to rural Ethiopia [7,8].

## Materials and methods

### Study setting, design and period

The study was conducted at Selam Health Center in Addis Ababa, Ethiopia—a central city (9°2'N, 38°45'E, elevation: 2446 m) covering 540 km² with over 5.7 million inhabitants [10]. Established in 1946 to serve underserved communities, the center manages about 200 daily emergency and outpatient visits. Its antenatal care department provides prenatal check-ups, screenings, health education, and counseling through a multidisciplinary team in dedicated, well-equipped facilities [11]. This study was conducted at Selam Health Center, one of the busiest maternal and child health facilities in Addis Ababa, Ethiopia’s capital city.

Addis Ababa is home to a highly diverse population, including women from different ethnic groups, educational levels, and socioeconomic backgrounds. The city has relatively better healthcare access compared to rural areas, yet nutritional challenges such as poor dietary diversity and urban poverty remain prevalent. Selam Health Center serves a wide catchment area, providing antenatal care to women from both low‑income households and middle‑income families, making it an ideal site to capture the sociodemographic determinants of iron deficiency anemia in an urban Ethiopian context. By situating the study in Addis Ababa, our findings complement national prevalence data with localized evidence that reflects the realities of Ethiopia’s growing urban population, thereby strengthening the relevance of our results for public health policy and intervention planning [8,9]. *A cross-sectional institutional study was conducted from April to May, 2024.*

### Study population and selection criteria

The study included all pregnant mothers attending Selam Health Center during the research period as a source population, with a focus on those presenting for their first antenatal care (ANC) contact during study period as a study population. Pregnant mothers attending their first antenatal care(ANC) visit who provided informed consent were included, while those on iron therapy or with chronic infections/inflammation (CRP positive) were excluded.

### Sample size calculation and sampling technique

The sample size was calculated based on an 11.3% IDA prevalence from a similar Ethiopian study [12], using standard statistical methods (95% CI, Z = 1.96, d = 0.05), which yielded 154 participants. After adding a 10% non-response rate, the final sample size was approximately 170. First ANC pregnant mothers were selected using non-probability convenience sampling.

### Variables of the study

The dependent variable was the magnitude of iron deficiency anemia. The independent variables included socioeconomic and demographic factors (age, occupation, education, residence, family size), clinical factors (intestinal parasitic infections, morning sickness, chronic infections, dental problems), obstetric and gynecological factors (history of abortion, gravidity, trimester), and nutritional factors (number of meals per day, mid-upper arm circumference, dietary diversity).

### Measurements and data collection

Structured questionnaires were prepared, translated into local languages, and administered through personal interviews, with supplementary clinical data from patient records. The structured questionnaire used for data collection was adapted from previously published studies on maternal nutrition and anemia. To ensure validity and reliability, the questionnaire was translated into Amharic and back‑translated into English. It was pre‑tested on 5% of the sample size at a nearby health center not included in the study, and necessary modifications were made based on feedback. Internal consistency was assessed using Cronbach’s alpha, which demonstrated acceptable reliability. These steps ensured that the questionnaire was culturally appropriate, clear, and capable of accurately capturing the sociodemographic and dietary information required.

Nutritional status was assessed using the Mid-Upper Arm Circumference (MUAC) measured at the arm’s midpoint, with cut-offs of <23 cm (underweight) and >29.5 cm (obesity) in pregnancy [13]. Nutritional status was assessed using mid‑upper arm circumference (MUAC). A cutoff of <23 cm was used to define under nutrition, while MUAC ≥23 cm was considered normal, consistent with WHO recommendations for pregnant women ([14–16]. This cutoff has been widely applied in maternal nutrition studies to identify women at risk of poor pregnancy outcomes [17].

Participants with elevated C‑reactive protein (CRP) levels were excluded to minimize confounding from inflammation. We defined CRP positivity as values greater than 5 mg/L, consistent with WHO and international reference standards for identifying acute inflammation [15,18,19]. This cutoff was selected because inflammation can alter iron metabolism and mask true iron deficiency anemia, thereby ensuring that our analysis focused on non‑inflamed individuals [7].

Dietary diversity was evaluated via a 24-hour recall based on FAO 2019 guidelines, with the score calculated from 10 food groups [20,21]. Dietary Diversity Score (DDS) was assessed using the FAO guidelines based on the number of food groups consumed in the previous 24 hours [21]. Adequate DDS was defined as consumption of ≥5 food groups, while inadequate DDS was defined as consumption of <5 food groups, consistent with the minimum dietary diversity for women indicator [22]. This cutoff is widely used in maternal nutrition studies and reflects minimum dietary diversity for women [23].

Specimens collected from the ANC department included 4 ml of morning venous blood (split equally between EDTA and SST tubes) and stool samples. Ferritin and hemoglobin levels were measured using the Cobas-2600 and Mindray CBC analyzers respectively, while stool samples were examined for intestinal parasites and C-reactive protein levels were screened by trained laboratory professionals.

Anemia was defined according to WHO criteria, with hemoglobin (Hb) thresholds adjusted for pregnancy trimester: Hb < 11 g/dL in the first trimester, Hb < 10.5 g/dL in the second trimester, and Hb < 11 g/dL in the third trimester [15]. Iron deficiency anemia (IDA) was defined as anemia in combination with serum ferritin <15 µg/L in the absence of inflammation (CRP ≤ 5 mg/L), consistent with WHO and international reference standards ([18,19]. These cutoffs are consistent with international standards and ensure accurate classification of anemia and IDA among pregnant women [7].

### Data quality assurance

Data quality assurance was ensured through a comprehensive process that integrated data collection and laboratory testing protocols. Each questionnaire was assigned a unique ID that corresponded to its specimen ID, and daily cross-checking was conducted to verify the completeness and accuracy of the collected information. In the laboratory, strict adherence to Standard Operating Procedures (SOP) and Internal Quality Control (IQC) guidelines guaranteed the quality of test results. During the pre-analytical phase, standard procedures were followed for sample collection, transportation, and processing; in the analytical phase, quality control materials were tested before sample analysis and only proceeded when the control tests met the required standards using updated materials provided by manufacturers and handled by trained laboratory professionals (e.g., hematological controls with the Mindray BC 3000 Plus were used to verify hemoglobin accuracy, with similar protocols for ferritin and C-reactive protein tests). In the post-analytical phase, findings were documented clearly in legible handwriting on data collection sheets and interpreted according to established reference ranges, thus ensuring both data integrity and the reliability of laboratory results.

### Data interpretation and analysis

The collected data was entered into SPSS version 27 and analyzed using both bi-variable and multivariable logistic regression to identify factors associated with IDA. Variables with a p-value less than 0.25 in the bi-variable tests were selected for the multivariable analysis to account for potential confounding, and the model’s goodness of fit was assessed using the Hosmer and Lemeshow approach. Adjusted odds ratios (AOR) with 95% confidence intervals were calculated to determine the strength of the associations, and all statistical tests were two-tailed with a significance level set at p < 0.05. The findings were then presented through narrative descriptions and descriptive statistics, including tables.

### Ethical conduct approval

In compliance with the Helsinki Declaration of Medical Research Ethics, this study was conducted with full ethical oversight. The Department Research and Ethical Review Committee (DRERC) of the Medical Laboratory Sciences Department, College of Health Sciences, Addis Ababa University, granted ethical approval for this research under protocol number DRERC/757/24/MLS/. All participants provided written informed consent after receiving detailed information about the study’s objectives and procedures. Confidentiality was safeguarded by assigning coded identifiers to participants, and no unauthorized individuals had access to the data. A total of 200 pregnant women were approached for participation. Of these, 185 provided written informed consent. Fifteen were excluded due to elevated CRP levels, incomplete data, or not meeting inclusion criteria. The final sample size was 170 eligible participants. This process ensured that only women meeting the study criteria were included, minimizing bias and maintaining data quality.

## Results

### Socio-demographic characteristics

Out of the 170 participants in the research, the majority, 111 individuals (65.3%), were aged between 15 and 24 years. In terms of education, 149 participants (87.6%) were having a formal education. The data indicated that most participants, 153 individuals (90%), lived in urban environments (Addis Ababa), while the rest were situated in rural area (sululta, and sansusi oromia regions). Furthermore, 125 participants, or 73.5%, belonged to families with more than five members (Table 1).

**Table 1:** Socio-demographic characteristics of study participants in Selam Health Center, Addis Ababa, Ethiopia, 2024 (n = 170).

Variables	Categories	Frequency	Percentage (%)
Age (yrs.)	15–24	39	22.9
	25–34	111	65.3
	> 35	20	11.8
Occupation	Government employee	15	8.8
	Private employee	45	26.5
	Merchant	5	2.9
	Housewife	89	52.4
	Daily laborer	6	3.5
	Student	10	5.9
Education	Informal	21	12.4
	Formal	149	87.6
Residence	Rural	17	10
	Urban	153	90
Family size	< 5	125	73.5
	> 5	45	26.5

### Obstetrics factors and gynecological characteristics

In our study, it was found that 128 participants, representing 75.3%, possessed information regarding anemia. Additionally, 123 participants, or 72.4%, were classified as multigravida. Among the participants, 39 individuals, which is 22.9%, reported a history of abortion within the last year, and 121 participants, equating to 71.2%, were in their first trimester of pregnancy (Table 2).

**Table 2:** Obstetrics and gynecological factors among pregnant women in Selam Health Center, Addis Ababa, Ethiopia, 2024 (n = 170).

Variables	Categories	Frequency	Percentage
Previous history of abortion (in the last year)	Yes	39	22.9
	No	131	77.1
Information about anemia	Yes	128	75.3
	No	42	24.7
Have child before this pregnancy	Yes	111	65.3
	No	59	34.7
Number of children	< 2	144	84.7
	> 2	26	15.3
Morning sickness	Never	50	29.4
	Rarely	44	25.9
	Sometimes	38	22.4
	always	38	22.4
Gravidity	Primigravida	47	27.6
	multigravida	123	72.4
Trimester	First	121	71.2
	Second	20	11.8
	Third	29	17.1

### Nutrition-related characteristics and feeding habit

In our study, the majority of the participants, totalling 126 (74.1%), had access to dietary information. Out of the 170 pregnant women included in the study, 145 (85.3%) reported eating meals three or more times a day, in contrast to 25 (14.7%) who consumed fewer than three meals daily. Among the participants, 45 (26.5%) were identified as having food allergies. Additionally, only 11 participants (6.5%) had medical conditions that affect their eating habits. The analysis also revealed that 36 participants (21.2%) consumed a limited variety of foods, defined as fewer than five food groups, whereas 134 participants (78.8%) exhibited a sufficient diversity in their dietary intake (Table 3).

**Table 3:** Nutrition-related characteristics and feeding habit among pregnant women in Selam Health Center, Addis Ababa, Ethiopia, 2024 (n = 170).

Variables	Categories	Frequency	Percentage (%)
Having dietary information	Yes	126	74.1
	No	44	25.9
Food allergy	Yes	45	26.5
	No	125	73.5
Medical condition that affects eating habit	Yes	11	6.5
	No	159	93.5
Dental problem that affects eating habit	Yes	14	8.2
	No	156	91.8
Meals consumed per day	Less than three	25	14.7
	Three and more	145	85.3
dietary diversity score (DDS)	Adequate	134	78.8
	Inadequate	36	21.2

### Hematological profile among first ANC pregnant mothers

In our study, pregnant mothers diagnosed with IDA exhibited significantly lower mean values for RBC, HCT, Hgb, and MCH compared to pregnant mothers without IDA. Specifically, the mean RBC value was 3.12 ± 0.36 versus 4.13 ± 0.43 (p < 0.001), the mean HCT value was 27.60 ± 3.67 versus 37.97 ± 3.58 (p < 0.001), the mean Hemoglobin value was 9.40 ± 1.11 versus 12.35 ± 1.12 (p < 0.001), and the mean MCH value was 23.50 ± 2.69 versus 30.29 ± 2.16 (p < 0.001). On the contrary, pregnant mothers with IDA had a higher mean RDW value (16.11 ± 0.91) compared to those without IDA (14.35 ± 0.69, p < 0.001) (Table 4).

**Table 4:** Hematological profile of first ANC pregnant women at selam health center, Addis Ababa, Ethiopia, 2024 (n = 170).

Variables	IDA	Not IDA	P-value
	Mean ±SD	Mean ±SD	
Red blood cell (cells/µL)	3.12 ± 0.36	4.13 ± 0.43	**< 0.001**
Hemoglobin(g/dL)	9.40 ± 1.11	12.35 ± 1.12	**< 0.001**
Hematocrit (%)	27.60 ± 3.67	37.97 ± 3.58	**< 0.001**
Mean Cell Volume (fL)	88.24 ± 1.63	92.11 ± 5.69	0.075
Mean Corpuscular Hemoglobin (pg)	23.50 ± 2.69	30.29 ± 2.16	**< 0.001**
Mean Corpuscular Hemoglobin Concentration (g/dL)	33.07 ± 0.93	32.28 ± 1.22	0.093
Red cell Distribution Width (%)	16.11 ± 0.91	14.35 ± 0.69	**< 0.001**

### Magnitude of iron deficiency anaemia

The analysis showed that anemia prevalence was 9.4% (16 out of 170 participants), with a particular emphasis on iron deficiency anemia, which affected 4.1% (7 out of 170 participants). Furthermore, 34 participants, representing 20%, were classified as undernourished, as indicated by a Mid-Upper Arm Circumference (MUAC) measurement of less than 23 cm. additionally, 16 women (9.4%) exhibited low hemoglobin levels, qualifying them as anemic, while 7 participants (4.1%) displayed low ferritin levels indicative of iron deficiency anemia. The stool examinations revealed that 9 participants (5.35%) were infected with intestinal parasites, including 11.1% with Hymenolepis *nana*, 33.3% with Giardia *lamblia*, and 55.6% with Entamoeba *histolytica* (Table 5).

**Table 5:** Clinical-related and other factors among pregnant women in Selam Health Center, Addis Ababa, Ethiopia, 2024 (n = 170).

Variables	Categories	Frequency	Percentage (%)
MUAC (cm)	23 cm and above	136	80
	Less than 23 cm	34	20
Hemoglobin (Hg) level (g/dl)	Low	16	9.4
	Normal	154	90.6
Ferritin level (µg/L)	Low	7	4.1
	Normal	163	95.9
Intestinal parasite infection	Negative	161	94.7
	Positive	9	5.3
Iron deficient anaemia/IDA	Yes	7	4.1
	No	163	95.9

*Footnote: MUAC (Mid-Upper Arm Circumference) measurements were utilized to assess the nutritional status of pregnant mothers. Participants were classified as undernourished if their MUAC was less than 23 cm, while values between 23 cm and 28 cm were considered normal, and values greater than 28 cm indicated obesity. Hemoglobin levels were categorized as anemic when below 11 g/dL, with values above this threshold considered normal. Ferritin levels were regarded as low and indicative of iron deficiency if they were below 15 µg/L, while values above this level were deemed normal.*

The bivariate logistic regression analyses were performed to identify the independent factors of IDA among expectants. The factors like low MUAC, less frequency of meals per day, previous history of abortion, and low dietary diversity score were found to be candidates for multivariate logistic regression (Table 6).

**Table 6:** Bivariate binary logistic regression analysis on factors associated with iron deficiency anemia (IDA) among pregnant women in Selam Health Center, Addis Ababa, Ethiopia, 2024 (n = 170).

Variables	Categories	IDA		COR (95%CI)	P-value
		Yes (n = 7)	No (n = 163)		
Information about anemia	Yes	4	124	1	0.271
	No	3	39	2.39 (0.51-11.12)	
Child before	Yes	4	107	0.698 (0.15-3.23)	0.645
	No	3	56	1	
Number of children	< 2	5	139	1	
	> 2	2	24	2.32 (0.43-12.63)	0.332
Family size	< 5	4	121	1	0.326
	> 5	3	42	2.16 (0.46-10.05)	
Gravidity	Primigravida	2	45	1	
	multigravida	5	118	0.95 (0.18-5.09)	0.955
Trimester	First	2	119	1	
	Second	2	18	6.61 (0.88-49.92)	0.670
	Third	3	26	6.87 (1.09-43.18)	0.40
Having dietary information	Yes	4	122	1	0.306
	No	3	41	2.23 (0.48-10.39)	
Food allergy	Yes	3	42	2.16 (0.47-10.05)	0.326
	No	4	121	1	
Chronic infection or continues medication that affects eating habit	Yes	1	10	2.55 (0.28-23.28)	0.407
	No	6	153	1	
Dental infection that affects eating habit	Yes	1	13	1.92 (0.22-17.21)	0.559
	No	6	150	1	
MUAC (cm)	23 and above	2	134	1	
	< 23	5	29	11.6 (2.14–62.5)	0.005 ^**a**^
Frequency of meal per day	< 3 times	3	22	4.8 (1-22.94)	
	> 3 times	4	141	1	0.049 ^**a**^
Previous history of abortion	Yes	4	35	4.9 (1.04 −22.8)	
	No	3	128	1	0.044 ^**a**^
Dietary diversity score (DDS)	Adequate	2	132	1	0.006 ^**a**^
	Inadequate	5	31	10.65 (1.97 −57.45)	

^**a**^ = variables exported to multivariate logistic regression; 1 = reference groups.

In the multi-variable logistic regression assessment, factors such as MUAC, frequency of meals consumed per day, and DDS were found to be statistically significant in relation to IDA, indicated by a p – value below 0.05, and a 95% CI (Table 7).

**Table 7:** Multivariate analysis of variables associated with iron deficiency anemia (IDA) among pregnant women in Selam Health Center, Addis Ababa, Ethiopia, 2024 (n = 170).

Variables	Categories	IDA		COR (95%CI)	AOR (95%CI)	P -value
		Yes (n = 7)	No (n = 163)			
MUAC (cm)	23 and above	2	134	1	1	
	< 23	5	29	11.6 (2.14–62.5)	9.84 (1.60–60.46) *	**0.014**
Frequency of meal per day	< 3 times	3	22	4.8 (1-22.94)	4.98 (1.79–31.33) *	
	> 3 times	4	141	1	1	**0.047**
Previous history of abortion	Yes	4	35	4.9 (1.04 −22.8)	1.87 (0.32 −11.09)	
	No	3	128	1	1	0.491
Dietary diversity score (DDS)	Adequate	2	132	1	1	**0.023**
	Inadequate	5	31	10.65 (1.97 −57.45)	9.13 (1.36-61.48) *	

MUAC – Middle upper arm circumstance, *P < 0.05 and 95% CI = confidence interval. 1 = reference groups.

A pregnant woman with a MUAC under 23 cm were 9.84 folds considerably more susceptible to the development of IDA relative to MUAC measurements of 23 cm or above (AOR = 9.84; CI: 1.60–60.46). This investigation revealed that pregnant women who ate fewer than three meals per day and had a low dietary diversity score had 4.98 and 9.13 increased likelihood of developing IDA in comparison to those who consumed three or more meals per day and had an adequate DDS (AOR = 4.98; CI: 1.79–31.33; AOR = 9.13; CI: 1.36–61.48), respectively (Table 7).

## Discussion

### Prevalence of iron deficiency anaemia

Among micronutrient deficiencies, iron deficiency stands out as the most frequently occurring globally [24,25] and is acknowledged as the leading cause of anaemia, accounting for roughly 25–50% of all anaemia cases. According to a report by the WHO, nearly half of all anaemic pregnant women suffer from IDA, a finding that aligns with the results of this study [26,27]. Our study reveals that the overall prevalence of anemia is 9.4%, with iron deficiency anemia accounting for 4.1% of this figure. In contrast, a cross-sectional study conducted by Gebreweld A *et al*. in 2018 and Terefe B *et al*. in 2015 at St. Paul Hospital reported anemia and iron deficiency anemia prevalence of 11.9% and 23.6%, respectively. Their analysis showed that pregnant women in the second and third trimesters had a significantly higher likelihood of experiencing anemia compared to those in their first trimester [28]. This discrepancy may explain why our findings are lower, as the majority of participants in our study were in their first trimester.

Our study finding was comparable to those of Enawgaw B *et al* in 2019, who reported an overall prevalence of iron deficiency anaemia at 3.2% (7 out of 217 subjects) [29]. Additionally, our research aligns with the findings done in 2021 by Pobee RA *et al*, in Ghana which reported a 6% incidence of IDA. Their work indicated that the extent of IDA was more pronounced during the second and third trimesters in comparison to the first trimester [30]. These factors may be relevant to our investigation, where the lower prevalence observed can be attributed in light of the truth that most of our study units were in their first trimester.

On the other hand, the prevalence of iron deficiency anaemia observed in our investigation is somewhat lower from the findings noted in 2014 by Gebremedhin S et al. in rural Sidama, Southern Ethiopia, as well as those reported by Laelago et al. in Wolayita Sodo, Ethiopia, where the rates were found to be 8.7% [31] and 11% [12], respectively.

The differences noted can be ascribed to various socio-demographic factors, nutritional habits, and variations in healthcare access, sample size differences, and the occurrence of other diseases that may lead to IDA. The lower incidence of IDA observed may be due to most participants living in urban settings with better nutritional access. These urban settings may foster a more supportive environment for the health and nutritional well-being of the participants. Improved dietary conditions can enhance iron bioavailability, which may influence the prevalence of IDA during pregnancy [32].

### Associated risk factor with iron deficiency anaemia

The nutritional status of women demonstrated statistically important connection with the initiation of IDA. Women presenting with MUAC of less than 23 cm, indicative of inadequate nutritional status, were found to be 9.84 folds more probable to develop IDA in contrast to their counterparts. This situation may arise from under nutrition, which leads to impaired iron absorption, and this under nutrition is attributed to a deficiency in the consumption of iron-rich foods [33]. The findings of our research align with the studies conducted by Kube OT et al. in 2016 in Kenya [34], as well as investigations in Bahrain [35]. Additionally, a study by Annan RA et al. in 2021 in the Ashanti Region, Ghana indicated an association between MUAC and the prevalence of iron deficiency anaemia. Specifically, individuals with reduced MUAC measurements were more predisposed to developing IDA [27].

There is a significant association between IDA and dietary diversity scores and frequency of meals per day. Pregnant women with inadequate dietary diversity scores demonstrated 9.13 times more likely to develop IDA in contrast to those with adequate dietary diversity scores. Furthermore, those who consumed fewer than three meals daily were 4.98 folds more probable to develop IDA in contrast to had three or more meals.

The physiological demands of pregnancy necessitate a significantly elevated nutritional intake, as the nutritional requirements increase substantially to support both the pregnant individual and the developing fetus. A common issue observed is the inadequate consumption of dietary iron, leading to insufficient iron levels in the foods consumed. As a result, there is a decline in overall iron levels, and these lead to developing IDA. This conclusion corresponds with the outcomes of investigations performed in jigjiga, Somali region and Northern Ghana [36,37].

A research investigation carried out in Kenya revealed a noteworthy finding positive linear correlation between dietary diversity and maternal IDA [38]. In a study by Laelago *et al*. in 2023 in the Wolayta Zone of Southern Ethiopia, it was found that pregnant women with an inadequate dietary diversity score were 5.2 folds more likelihood to develop IDA opposed to those with an adequate score [12]. Additionally, a study by Diana *et al*. in 2019 in Indonesia highlighted that a less diversified diet, particularly one deficient in dietary iron, was a contributing factor to IDA among pregnant women [39]. Similarly, an investigation executed by Annan RA *et al*, 2021 among the expectants in Shanti Region of Ghana found that inadequate DDS were more prone to depleted body iron stores and the development of IDA compared to their counterparts with adequate scores [40].

Numerous studies indicate that the diverse dietary habits of pregnant women can significantly affect their iron levels, potentially resulting in IDA during pregnancy. A study conducted in Westmoreland, Jamaica, [41] along with a study by Burayu ET and Degefa BD, [42] in southwestern Ethiopia, demonstrate that a pregnant women who include green leafy vegetables in their diets develop a lower risk of developing IDA in contrast to don’t consume such edible plants. This indicates that consuming a variety of diets may enhance iron levels, thereby decreasing the risk of developing IDA. It is essential to continue educating pregnant women on the significance of incorporating more iron-rich foods into their diets while minimizing or limiting the intake of foods that hinder iron absorption.

### Limitation of the study

This study has several limitations. First, it was conducted at a single health center in Addis Ababa, which may limit the generalizability of the findings to other regions of Ethiopia, particularly rural areas with different sociodemographic and dietary patterns. Second, the relatively small sample size may reduce statistical power and increase the risk of type II error. Third, the cross‑sectional design restricts causal inference, as associations observed cannot establish temporal relationships. Finally, although efforts were made to exclude participants with inflammation using CRP, residual confounding factors may still influence iron status. Despite these limitations, the study provides important baseline evidence on the magnitude and determinants of iron deficiency anemia among first ANC attendees in an urban Ethiopian setting.

## Conclusion and recommendations

This study demonstrated that iron deficiency anemia (IDA) remains a significant public health problem among first antenatal care attendees at Selam Health Center, Addis Ababa. The magnitude of IDA was influenced by sociodemographic and nutritional factors, including dietary diversity and maternal nutritional status. These findings highlight the importance of early screening and intervention during the first ANC visit.

We recommend strengthening maternal nutrition education, promoting dietary diversity, and ensuring timely iron supplementation programs at the community and health‑facility level. Further multicenter studies with larger sample sizes are needed to validate these findings and provide nationally representative data to guide maternal health policies in Ethiopia.
